# Type 1 diabetes prevention and treatment: Time to think outside the box

**DOI:** 10.1111/1753-0407.13502

**Published:** 2023-11-13

**Authors:** Marcelo Maia Pinheiro, Felipe Moura Maia Pinheiro

**Affiliations:** ^1^ UNIVAG, Centro Universitário Várzea Grande Brazil; ^2^ Faculdade de Medicina de São Paulo Hospital das Clínicas São Paulo Brazil

Dear Editor,

Atkinson and Mirmira^1^ point out that the actual abnormalities in type 1 diabetes (T1D) lie beyond the three classic seminal observations of inflammatory infiltrate of islets, genetic susceptibility associated with the major histocompatibility complex, and autoantibodies against β‐cell antigens. Indeed, many other abnormalities are present in T1D, including dysfunction of α cells, abnormalities of the exocrine pancreas, sympathetic islet neuropathy, and hemorrhages within the islets.^1^, ^2^

Given this, it seems obvious that strategies to prevent and treat T1D based on autoimmunity alone are doomed to fail over time. Studies in T1D using monotherapies acting on a single pathway of the immune system, as teplizumab,^3^ may delay the decline in C‐peptide levels but do not appear to fundamentally alter the underlying pathophysiology of the disease. We believe that therapies aimed at interrupting the progression of T1D should act on both the immune system and the islets by improving α and β‐cell dysfunction. Ashraf et al^4^ published a meta‐analysis showing that anti‐CD3 antibody treatment increases endogenous insulin production and reduced insulin dosage without improvement in HbA1c when compared with placebo. The T1D‐modifying agent, as teplizumab should not only preserve the C‐peptide concentration, but also prolong the honeymoon phase.

Our group published a study with T1D showing that treatment with sitagliptin plus vitamin D3 prolonged the honeymoon period, resulting in some participants being insulin free for up to 24 months.^5^ Yan et al^6^ and Zhang et al^7^ in clinical trials in China showed positive results in adults with T1D treated with saxagliptin plus vitamin D3. Another association that has shown promising results on T1D in nonobese diabetic mice and human studies is the combination of dipeptidyl peptidase 4 (DPP‐4) inhibitors, gamma‐aminobutyric acid, and proton‐pump inhibitor (PPI), resulting in long insulin‐free periods.^8^, ^9^ Reddy et al^10^ showed a smaller decline in residual β‐cell function and lower insulin requirements in T1D treated with lansoprazole plus cholecalciferol. Verapamil has been shown to delay the progression of T1D for at least 2 years after diagnosis, partially preserved stimulated C‐peptide secretion, normalizing the serum levels of chromogranin A (a T1D autoantigen), proinflammatory interleukin‐21, and T–follicular‐helper cell markers.^11^, ^12^ Verapamil also regulates the thioredoxin system and promotes an antioxidative, antiapoptotic, and immunomodulatory gene expression profile in human islets.^11^

We have a long way to go before we can identify the best combinations (if double, triple, or even more drugs) and dosages of these drugs. The DPP‐4 activity appears to be higher in patients with T1D than in those with T2D and normal controls.^2^ Does this mean that patients with T1D require higher doses of DPP‐4 inhibitors? The same question applies to PPI. Griffin et al^13^ addressed this issue in their study of patients with new‐onset T1D treated with sitagliptin and lansoprazole (REPAIR‐T1D). In their study, the increases in glucagon‐like peptide‐1 and gastrin concentrations expected with the conventional dose used for each drug were not observed in all the participants.

In conclusion, trials on drug repositioning using combinations of medications acting on the immune system and on islets are still needed to prove this theory.

## AUTHOR CONTRIBUTIONS

The authors contributed equally in preparing and reviewing this letter and approved its final version for publication.

## FUNDING INFORMATION

None.

## DISCLOSURE

No potential conflicts relevant to this article were reported.
